# Absorption of N-acetylcysteine in Healthy and *Mycoplasma gallisepticum*-Infected Chickens

**DOI:** 10.3390/vetsci8110244

**Published:** 2021-10-20

**Authors:** Tsvetelina Petkova, Aneliya Milanova

**Affiliations:** Department of Pharmacology, Animal Physiology, Biochemistry and Chemistry, Faculty of Veterinary Medicine, Trakia University, 6000 Stara Zagora, Bulgaria; akmilanova@gmail.com

**Keywords:** poultry, N-acetylcysteine, pharmacokinetics

## Abstract

N-acetylcysteine (NAC) is widely used as a mucolytic agent in cases with inflammation of the lungs. NAC is applied in poultry with aflatoxin B1 intoxication as an antioxidant, but its pharmacokinetics are not known. The present study was conducted to characterize the population pharmacokinetics of orally administered NAC in broilers. It included 32 chickens, divided into four groups, treated with NAC at a dose rate of 100 mg/kg/day mixed with the feed: healthy broilers (*n* = 6); chickens infected with *Mycoplasma gallisepticum* (*n* = 10); healthy broilers (*n* = 6); and diseased chickens (*n* = 10) treated with NAC and doxycycline (via drinking water, 20 mg/kg body weight (b.w.)). Plasma concentrations were analyzed by Liquid Chromatography –Mass Spectrometry (MS)/MS. NAC was absorbed after oral administration in all four groups of chickens. In healthy chickens treated solely with NAC, maximum plasma concentrations of 2.26 ± 0.91 µg mL^−1^ were achieved at 2.47 ± 0.45 h after dosing. The value of absorption half-life was 1.04 ± 0.53 h. The population pharmacokinetic analysis showed that dose adjustment of NAC is not required in *M. gallisepticum*-infected broilers or when it is combined with doxycycline.

## 1. Introduction

Acetylcysteine (NAC) is the N-acetyl derivative of the amino acid L-cysteine, and it is a precursor of glutathione in the body. The thiol group is able to reduce free radicals and has antioxidant effects [1,2,3,4]. Its mode of action substantiates its application in the treatment of intoxication with drugs and heavy metals [5]. NAC was found to have antibacterial and antibiofilm activity against micro-organisms such as *Staphylococcus aureus*, *Pseudomonas aeruginosa*, and other pathogenic bacteria [6,7]. N-acetylcysteine (NAC) possesses mucolytic activity and is often used in humans in the treatment of chronic bronchitis and other lung disorders, such as lung inflammation, transplant rejection, and pulmonary fibrosis [8,9]. NAC is applied in veterinary medicine with similar indications as in human medicine [10,11,12]. In the published literature, the efficacy of NAC in the treatment of stress and disease conditions in poultry is described [13]. The antioxidant properties of NAC substantiate its efficacy in the reduction of the toxic effects of aflatoxin B1 in broiler chickens and rabbits with experimental aflatoxicosis [14,15,16]. A positive effect in reducing the negative impact of heat stress in poultry was described [13,17]. 

Mycoplasmosis, caused by *Mycoplasma gallisepticum* (MG), is a global respiratory disease that affects poultry and leads to a reduction in the laying capacity and growth of chickens [18]. MG provokes chronic inflammation of the airways and lungs, respiratory distress, severe inflammation of the mucous membranes, and lesions of the trachea and air sacs of varying severities [19,20]. MG evades the host immune system of chickens and causes immune dysregulation, which allows other infectious diseases to develop [20,21]. The infection disrupts normal homeostasis in the cells causing mitochondrial damage and increased production of reactive oxygen species [20,22]. Knowledge about the mechanism of action of NAC suggests that it may have therapeutic value in MG infection, which persists in chickens for life. NAC is applied in chickens at a dose rate from 50 to 800 mg kg^−1^ body weight (b.w.); however, nothing is known about its pharmacokinetics in this animal species [14,17,23]. Complete pharmacokinetic investigations were performed in humans and there is one study concerning its absorption and disposition in cats [11,24]. Optimal dosing regimen of NAC in chickens requires information about its absorption after oral treatment via feed, which is the main route of drugs administration applied in poultry husbandry. 

Therefore, the aim of this study was to evaluate the absorption of NAC after oral administration with feed in healthy and *M. gallisepticum*-infected chickens through the population approach. Population pharmacokinetic analysis was used to reveal the possible effect of doxycycline, a widely used antibiotic against mycoplasmosis, on the absorption of NAC.

## 2. Materials and Methods

### 2.1. Drugs

The N-acetyl-L-cysteine used for the treatment of the poultry and for the analytical tests was HPLC grade (≥ 99%, Sigma-Aldrich, St. Louis, MO, USA). Doxycycline hyclate in a form of HydroDoxx® 500 mg/g oral powder (Huvepharma NV, Antwerpen, Belgium) was applied in groups treated with a combination of NAC and antibiotic. The analysis of NAC concentrations was performed using the following reagents: d-penicillamine (98–101%) (Sigma-Aldrich, St. Louis, MO, USA), dl-dithiothreitol for molecular biology, ≥98% (HPLC) (Sigma-Aldrich, St. Louis, MO, USA), and trifluoroacetic acid (99.5%) (Fisher Chemical, Fisher Scientific, Waltham, MA, USA). Acetonitrile OPTIMA®, LC/MS grade (Fisher Chemical, Fisher Scientific, Waltham, MA, USA), formic acid for mass spectrometry ~98% (Honeywell Fluka™, Seelze, Germany), and water for chromatography (LC-MS Grade, LiChrosolv®, Merck KGaA, Darmstadt, Germany) were used for the preparation of mobile phases.

### 2.2. Experimental Design

The experiments were carried out after obtaining ethical approval from Bulgarian Food Safety Agency (License 245/25.09.2019). Ross hybrid (Cornish ♀ x Plymouth Rock ♂) one-day-old broilers (*n* = 52) were obtained from “Zhuliv“ EOOD, Stara Zagora. At the beginning of the experiment, a higher number of chickens was included due to the expected mortality after infection with *Mycoplasma gallisepticum*. The animals were placed in two separate units in order to preserve the healthy chickens from contact with the infected ones. The chickens were accommodated in a Biobase unit at the Faculty of Veterinary Medicine, Trakia University. They were reared for 50 days according to the requirements of the species (Ordinance No. 20/1.11.2012 on the minimum requirements for protection and welfare of experimental animals and requirements for use, rearing, and/or their delivery). The poultry received feed without antibiotics and cocidiostats suitable for every stage of their development (Vladini Trading EOOD, Chirpan, Bulgaria). The composition of the feed during the treatment with NAC is given in Table S1 from the Supplementary data. They were treated prophylactically twice with Bioselet E according to the manufacturer instructions (Biovet AD, Pestera, Bulgaria) on day 11–15 and on day 21–25 at a dose rate of 0.3 mL L^−1^ and 0.5 mL L^−1^ water, respectively. The daily requirements for the humidity and temperature in the units were followed according to the age of the chickens. The animals received feed and water *ad libitum*. The light regimen was 18 h daylight and 6 h in the dark. The treatment of the chickens started at the age of 40 days and the pharmacokinetic study included 32 broiler chickens divided in four groups as described below. The animals were weighed once per day before the treatment and the consumption of the feed was registered on the day before the experiment and during the entire experiment. The chickens were treated for five consecutive days. On the sixth day, the broilers received feed and water without supplementation of either NAC (all groups) or doxycycline (group 2 and group 4), respectively. 

The first group (*n* = 6) and the second group (*n* = 6) were healthy broilers. The first group was treated with NAC only, at a dose rate of 100 mg kg^−1^/24 h. The feed consumption was registered every 12 h and the recalculated obtained mean dose was 41 mg kg^−1^ b.w./12 h. The same procedure was followed for the second group and the recalculated consumed mean dose was 47.5 mg kg^−1^ b.w./12 h. The second group received doxycycline hyclate at a dose rate of 20 mg kg^−1^/24 h with drinking water, which was freshly prepared every 12 h and the consumed amount was registered. 

Broiler chickens (*n* = 36) were challenged with *Mycoplasma gallisepticum*. They were infected with a bacterial suspension of *M. gallisepticum* R low strain (Pendik Veterinary Control Institute, Istanbul, Turkey) on the first day after hatching. In order to have enough infected experimental animals, 36 chickens were inoculated. A culture of *M. gallisepticum* 1.10^8^ CFU/mL was used to provoke infection. Every chicken received bacterial suspension intrasaccularly in the left (0.1 mL) and the right cranial thoracic air sacs (0.1 mL). 

Pharmacokinetic studies in infected animals were performed in 20 broilers divided into two groups. The third group (*n* = 10 chickens) was treated with NAC via the feed. The targeted dose was 100 mg kg^−1^/24 h. The feed consumption was controlled every 12 h. The consumed amount of NAC was recalculated and the obtained mean dose was 43.29 mg kg^−1^/12 h. Broilers from the fourth group (*n* = 10) received NAC with the feed at a targeted dose of 100 mg kg^−1^/24 h and doxycycline hyclate with drinking water at a dose rate of 20 mg kg^−1^. The feed consumption was controlled as in the third group and the recalculated obtained mean dose was 43.30 mg kg^−1^/12 h. Drinking water with the antibiotic was freshly prepared and the consumed amount was registered every 12 h. 

Blood samples were obtained from v. subcutanea ulnaris before treatment and thereafter at the following time intervals: 0, 0.5, 1, 2, 3, 4, 6, 9, 12, 14, 24, 120, 122, 124, 126, 132, 144, 152, 168, and 174 h. Blood samples (0.8 mL) from groups of healthy chickens were collected from every chicken at every time interval. Blood samples (0.8 mL) from groups of infected chickens were obtained from six broilers at every time interval from the third and the fourth group, respectively. The samples were placed in Eppendorf tubes containing heparin-sodium and centrifuged at 3,000 rpm for 10 min. Plasma was transferred to clean tubes and stored at −80 °C until analysis.

### 2.3. Determination of Plasma Concentrations by LC-MS/MS Analysis

Concentrations of NAC in plasma were analyzed by the LC-MS/MS method. Extraction of NAC from plasma samples was performed according to the method described by Buur et al. [11] with minor modification. Briefly, 200 µL dithiotheriol (46.3 mg·mL^−1^) and 200 µL penicillamine (100 µg mL^−1^) were added to 200 µL of plasma. Then, 200 µL of 10% trifluoracetic acid in acetonitrile was added to the mixture. Precipitated samples were vortexed for 30 s and then centrifuged for 10 min at 14 800 × g. The supernatant was filtered through 0.22 µm syringe filters, transferred into injection vials, and 5 µL were injected into the LC-MS/MS system. 

Chromatographic separation was achieved by use of a Poroshell 120 EC C18 column (4.6 mm i.d. × 100 mm, 2.7 µm, Agilent Technologies, USA). Mobile phase A consisted of 0.1% formic acid in LC-MS grade water and mobile phase B was 100% acetonitrile. The applied gradient elution program was 0–1 min (90% A, 10% B) and 1–8 min (90% A, 10% B). The flow rate was 0.4 mL min^−1^. The total run time was 8 min with a post-run of 4.5 min. The liquid chromatography (LC) system consisted of a 1260 Infinity II quaternary pump and a 1260 Infinity II Vial Sampler. The samples were analyzed with a triple-quadrupole mass spectrometer Agilent 6460c with Agilent Jet Stream (AJS) technology for the determination of NAC concentrations. The run conditions were optimized with Mass Hunter Optimizer (Agilent Technologies, Santa Clara, CA, USA). Positive ion mode was applied (Agilent Jet Stream ESI+). The other parameters were as follows: gas temperature: 250 °C; drying gas: (nitrogen) 6 L/min; nebulizer gas: (nitrogen) 35 psi; sheath gas: (nitrogen) 400 °C; sheath flow: 12 L/min; capillary voltage: 4000 V; nozzle voltage: 500 V; and dwell time: 200 ms. The qualifying and the quantifying ions for NAC were 164.08 *m*/*z* and 122.00 *m*/*z*, respectively. These ions for penicillamine were 149.96 *m*/*z* and 87.10 *m*/*z*, respectively. The LC-MS/MS method was validated for NAC using plasma samples from untreated chickens spiked at seven concentrations of NAC: 0, 0.25, 0.5, 1, 2.5, 5, 7.5, and 10 µg mL^−1^. The internal standard was used at a final concentration of 0.25 µg mL^−1^. The standard curve was linear (R2 = 0.9874) between 0.25 and 10 µg mL^−1^. The values of limit of detection (LOD) and limit of quantification (LOQ) were 0.09 and 0.30 µg mL^−1^, respectively [25]. The mean accuracy was 96.53 ± 12.00%. The value of intraday precision was 5.89% and interday precision was 11.67%.

### 2.4. Pharmacokinetic Analysis 

As a first step, the pharmacokinetic parameters after oral administration of NAC in healthy broilers (*n* = 6) were computed using one-compartmental analysis (Phoenix 8.3, Certara, St. Louis, MO, USA) for each chicken. The most suitable model was chosen according to the Akaike information criterion (AIC). The following parameters were calculated: k_ab_: absorption rate constant; k_el_: elimination rate constant; t_1/2ab_: half-life of absorption; t_1/2el_: elimination half-life after oral administration; C_max_: maximum plasma concentration; T_max_: time to reach maximum plasma levels; AUC_0→∞_: area under the concentration time curve. Information from the classical pharmacokinetic analysis was used as a basis for the population pharmacokinetic modeling.

Phoenix NLME version 8.3 (Certara, St. Louis, MO, USA) was used for the population pharmacokinetic analysis by applying a nonlinear mixed effects (NLME) model. A one-compartment model was selected on the basis of a visual inspection of the plots, the values of log likelihood (-2LL), and AIC. Parameterization involved the following: absorption rate constant (ka), volume of distribution (V), and elimination rate constant (k). Several scenarios for the determination of interindividual variability were tested, including the effect of co-variables such as body weight, health status (healthy or infected with M. gallisepticum), and administration of drugs (NAC alone or in combination with doxycycline). The likelihood ratio test (LRT) test was applied for the statistical evaluation of the goodness-of-fit of the models in order to compare the more complex models to the basic model; the critical value of the χ2 distribution considered for a given nominal risk was 0.05. The results of -2LL, LRT, the AIC, and the software model comparison tool demonstrated that the co-variables had no effect on the improvement of the model when data from all four groups were modeled. Therefore, a one-compartmental model without covariates was chosen for this set of data. The same procedures were followed when data from the groups of healthy and infected poultry, treated solely with NAC, were analyzed. This dataset was separately analyzed in order to better understand the origin of between-subject variability. The LRT test did not show a significant improvement of the model when health status was added as a co-variable, and again, the model without covariates was chosen.

The between-subject variability (BSV), which describes the biological variability, was described by an exponential model, and the pharmacokinetic parameters (k, V, and ka) for every subject (ith animal) were defined as follows:

k*_i_* = tv_k_ × Exp(η*_i_* ), (1)

where k*_i_* is the elimination rate constant for the *i*th animal, tv_k_ (also referred to as θ) is the population k, and η*_i_* (eta) is the deviation associated with the individual animal *i*th from the computed population value of tvk. The other two parameters were calculated with the same algorithm. Normal distribution of etas with a mean value of 0 and a variance (ω^2^) was assumed. Consequently, individual parameters and their etas could be correlated. A full variance–covariance omega matrix was applied. The variance (ω^2^) was converted into a coefficient of variation (CV%) by applying the following equation:

(2) $$\mathrm{CVk}(\%)=100\;\times \;\sqrt{\exp\left(\omega^{2}k\right)-1},$$

where ω^2^k is the variance of the elimination rate constant.

The shrinkage of random effects towards the means was estimated according to the following equation:

Shrinkage = 1 − SD(η*_j_*)/ω*_j,j,_* (3)

where SD(η*_j_*) is the empirical standard deviation of the *j*th (observation) η over all N_sub_ subjects, and ωj,j is the estimate of the population variance of the *j*th random effect, *j* = 1, 2, …, N, et al.

Multiplicative and combined (additive + multiplicative) residual error models were tested to describe the intraindividual variability. Finally, due to the very low stdev0 value (0.001 ng/mL) and by following the criteria described above, multiplicative (proportional) residual error was used:

Ct = f (θ, Time) × (1 + ε)(4)

where ε has a mean of zero and a variance σ^2^. The first order conditional estimation extended least squares (FOCE-ELS) methodology was used for analyses. It was based on minimizing an extended least squares objective function representing the FOCE approximation to the negative log of the marginal likelihood as a function of (θ, σ-standard deviation, ω). Censored data were not included in the modeling. Typical values (tv) and their associated standard errors (SE) and coefficient of variation as an indication of the precision of the estimate were computed using a simple approach. To evaluate the goodness-of-fit of the final model, various diagnostic plots, such as a visual predictive check, population predicted value based on population parameter estimates (PRED), individual predictive value based on an individual’s etas (IPRED) versus the dependent variable (DV), conditional weighted residuals (CWRES), and the fitting of the individual curves, were examined. 

## 3. Results

Mycoplasmosis was successfully provoked in groups of chickens challenged with *M. gallisepticum* and typical clinical signs of infection were registered. A pathological investigation of some of the infected broilers proved the presence of changes characteristic for the disease. Healthy animals did not show any signs of disease during the experiment. Infected broilers showed clinical signs of mycoplasmosis such as nasal discharge, dyspnea, decreased appetite, and growth retardation. A tendency for decreased appetite was observed in infected groups during the first 15 days of life. The animals from the infected groups had a statistically significantly lower body weight and a higher FCR was calculated at the end of the experiment in comparison to the healthy broilers. Disorders in breathing were observed in some of the infected chickens until the end of the experiment. Clinical signs of the disease were not found in the groups of healthy chickens. Dead animals from the infected groups were subjected to a pathological examination, which showed thickened serosa, local serofibrinous exudate to polyserositis of the air sacs, and fibrinous airsacculitis in the chest and abdominal cavity. Mortality was not observed among the healthy chickens.

**Figure 1:**
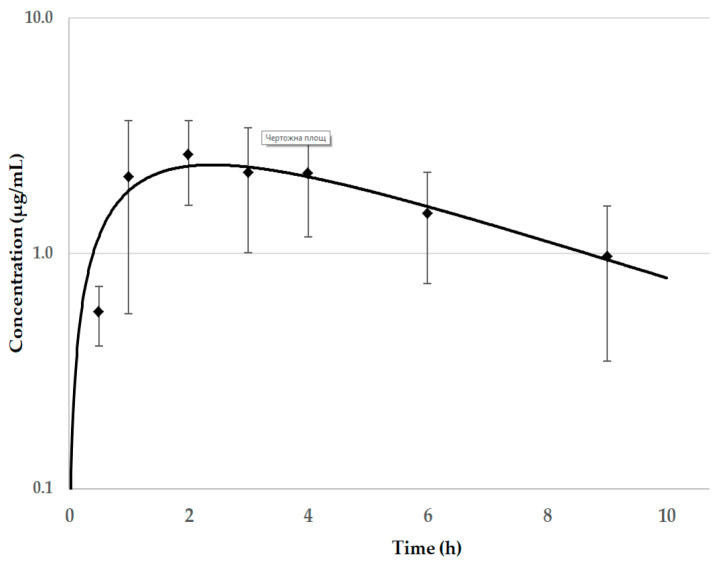
Semi-logarithmic mean ± standard deviation (SD) plasma (predicted levels: black line; ♦: observed concentrations) concentrations of N-acetylcysteine vs. time curve after oral administration in healthy chickens (*n* = 6). The received dose was 41 mg kg^−1^ body weight (b.w.)/12 h via feed (the proposed dose was 50 mg kg^−1^ b.w./12 h).

The analyzed plasma concentrations showed that NAC was absorbed after oral administration in healthy and in *M. gallisepticum*-infected chickens (Tables S2–S5). According to our knowledge, this is first pharmacokinetic study of NAC in chickens; therefore, complete data for plasma concentrations are given in the Supplementary Material. Plasma concentrations of NAC were detected at the first sampling point, i.e., at 0.5 h after the start of the treatment. Steady-state levels with some fluctuations were observed 12 h after the start of the treatment. The plasma concentrations were below the LOQ 2–4 h after the end of the treatment. Large differences in plasma levels between individual animals were found. The control of the consumed feed demonstrated that the received dose was very close to the proposed dose. One-compartmental analysis with absorption was used to calculate the pharmacokinetic parameters of NAC in the group of healthy chickens treated solely with this compound. C_max_ of 2.26 ± 0.91 µg mL^−1^ was detected at T_max_ of 2.47 ± 0.45 h (Table 1, Figure 1). The value of the absorption half-life was lower when compared to the elimination half-life. 

**Figure 2:**
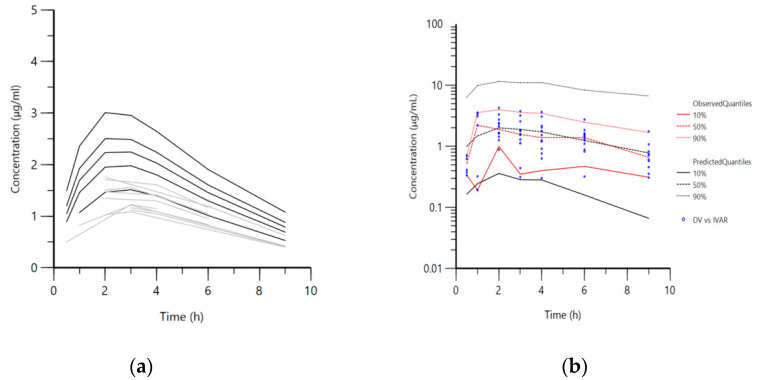
Groups treated solely with N-acetylcysteine. ((**a**)—left panel) Semi-logarithmic spaghetti plot of disposition curves of orally administered N-acetylcysteine in healthy (black lines) and *Mycoplasma gallisepticum*-infected chickens (gray lines) and ((**b**)—right panel) visual predictive check (VPC) plot depicting the observed quantiles (10, 50, and 90%) and corresponding predictive quantiles.

**Figure 3:**
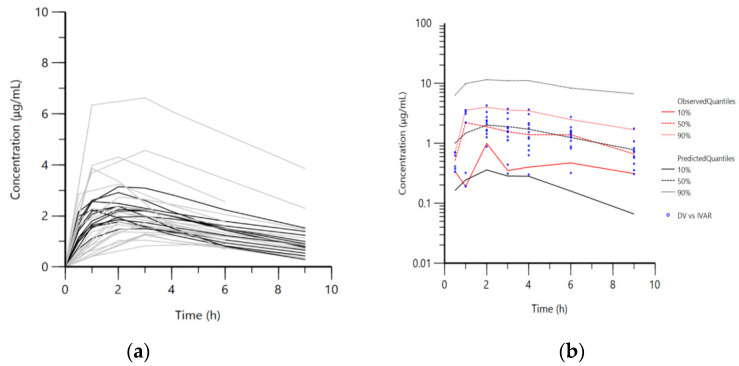
Data for all four groups. ((**a**)—left panel) Semi-logarithmic spaghetti plot of disposition curves of orally administered N-acetylcysteine in healthy (black lines) and *Mycoplasma gallisepticum*-infected chickens (gray lines) and ((**b**)—right panel) visual predictive check (VPC) plot depicting the observed quantiles (10, 50, and 90%) and corresponding predictive quantiles.

Further, a population pharmacokinetic analysis was performed with the data for the recalculated obtained doses from every group of chickens. A one-compartment model with first-order absorption and elimination was selected as the structural pharmacokinetic model (Figure 2a and Figure 3a). The visual predictive check plot (Figure 2b and Figure 3b) demonstrated that the selected model was appropriate to describe NAC disposition in healthy and infected broilers in all four groups and in the groups treated solely with NAC. 

**Figure 4:**
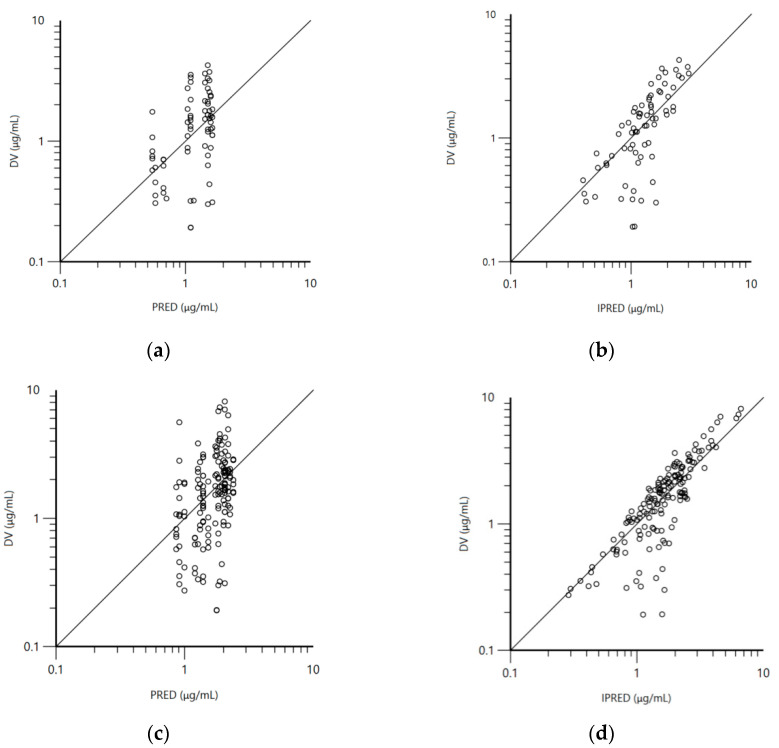
((**a**)—groups treated solely with N-acetylcysteine; (**c**)—data for all four groups) Logarithmic plot of the dependent variable (DV: observed N-acetylcysteine plasma concentrations) vs. population predicted N-acetylcysteine concentrations (PRED); ((**b**)—groups treated solely with NAC; (**d**)—data for all four groups) logarithmic plot of the dependent variable (DV: observed N-acetylcysteine plasma concentrations) vs. individual predicted N-acetylcysteine concentrations (IPRED).

A visual inspection of the goodness-of-fit plot of the population predicted concentration (Figure 4a,c) versus the observed NAC concentrations shows that the data are evenly distributed around the line of identity. The individual predicted concentrations of NAC were consistent with the observed data, with evenly distributed concentration values around the line of identity (Figure 4b,d). Figure S1a,b demonstrates that the values of conditional weighted residuals (CWRES) are grouped between y = −2 and y = 2. An inspection of the trend of the middle line showed that the data were evenly distributed around zero, and the absence of fanning indicated no major bias in the structural model.

Computed typical population mean values (θ) of the structural parameters of the population model, their standard errors, and the SD of the residuals are shown in Table 2. 

Table 3 presents the values of the random effects, the between-subject variability (BSV), and the shrinkage. The values of BSV were high and should be interpreted with caution when the dataset for all four groups was analyzed. The values of shrinkage for the dataset of all four groups were relatively close to the accepted breakpoint, i.e., 0.3, and when the results for the chickens treated solely with NAC were separately analyzed, more reliable data for BSV were obtained. The values of shrinkage for the dataset of groups of healthy and infected poultry, treated solely with NAC, were below 0.3, indicating no significant bias (Table 3).

## 4. Discussion

The current investigation attempted to characterize the pharmacokinetics of orally administered NAC in feed in healthy and *M. gallisepticum*-infected broilers through the population approach. One-compartmental analysis was applied to first characterize the pharmacokinetics of orally administered NAC in healthy broilers. The obtained data indicate that the pharmacokinetics of NAC are similar to those reported in humans and in cats. Low concentrations of NAC in the plasma of chickens and a very quick drop in plasma levels after the end of treatment can be explained by the rapid oxidation of the compound in the intestines and the extensive first-pass metabolism, as was observed in studies in humans [26]. The low bioavailability of NAC in cats was explained by a similar mechanism [11]. Differences in the drug dosage form, e.g., oral solutions, tablets (in cats and humans), and powder mixed with feed (in chickens), complicate the comparison of the pharmacokinetic parameters in these species. The value of C_max_ in chickens is almost 10 times lower than the reported a C_max_ of 19.66 ± 0.31 µg mL^−1^ in cats treated orally with 100 mg kg^−1^ NAC as an oral solution [11]. The applied drug formulations and the variations in feeding behavior of individual chickens can explain these differences. Similar to our results, repeated NAC administration in humans leads to significant variations in the plasma levels of the drug, which can be explained by differences between the subjects as regards absorption and/or metabolism of this agent [27]. Administration of 600 mg kg^−1^ as a tablet in healthy subjects resulted in a C_max_ of 2.5 ± 0.6 µg mL^−1^, which is very close to the value of C_max_ in healthy broilers. The T_max_ in cats was 0.85 ± 0.31 h and, in humans, it was between 1.4 ± 0.7 h and 1.9 ± 0.6 h [24,27,28]. The higher value of T_max_ in chickens indicates that a longer time is necessary to achieve maximum plasma levels, which can again be explained by the dosing route. Altogether, these results show that NAC is relatively quickly absorbed to a relatively low degree. No differences between single dose and repeated doses of NAC were observed in chickens, in line with the results in humans [24,29]. The values of elimination half-life in chickens (3.30 ± 1.43 h) differ from the data reported in humans, i.e., from 3.7 ± 0.8 to 15.4 ± 3.5 h in Chinese and 18.7 ± 7.2 h in Caucasian healthy subjects [24,28].

In the current study, the simplest model in terms of parameterization was selected for the population pharmacokinetic analysis with the intention of determining a possible effect of *M. gallisepticum* infection on the oral pharmacokinetics of NAC. The values of tvka were lower than the reported value of k01 1.58 ± 0.74 h^−1^ in cats, which may be attributed to the differences in the applied drug formulations [11]. The values of tvk, i.e., 0.51–1.219 h^−1^, were close to k10 of 0.81 ± 0.39 h^−1^ found in cats [11]. Lower values of k10 were observed in humans, i.e., from 0.187 to 0.04–0.045 h^−1^ [24,28]. Again, such comparisons should be made with caution due to differences in the dosing regimen and the drug dosage forms used. The effect of the disease condition as a co-variable was tested after observing the spaghetti plot from the dataset from poultry solely treated with NAC. The statistical criteria did not indicate an improvement in the population model by including a categorical co-variable, such as health status (healthy versus infected). Similarly, treatment, as a categorical co-variable (NAC treatment alone versus NAC co-administration with doxycycline), was not able to explain the source of variability. The data from the statistical tests were supported by a visual inspection of the plots for goodness-of-fit and by increasing the shrinkage values. On the basis of these results, the final model was built without the addition of any co-variables. The analysis of the dataset, including results from the groups treated solely with NAC, shows an acceptable value of shrinkage <0.30, and the VPC plot shows that less than 20% of the observed concentrations are outside the plotted quantiles. The values for BSV indicate small interindividual differences in absorption and the elimination rate of NAC. Contrarily, the goodness-of-fit plots for the dataset, including results from all four groups, should be read with caution due to high (>30%) values of shrinkage [30]. In this case, the values of BSV revealed that a high variability in the absorption, elimination, and volume of distribution corrected with bioavailability can be expected between individual animals. The increased bias when data from all four groups were analyzed indicates that the model requires further development. Presumably, another source of variability that was not identified in the current study would explain these results. In general, the data indicated that pharmacokinetics did not differ significantly between healthy and *M. gallisepticum*-infected broilers and that adjustment of the dose of NAC was not required in this disease condition. A more complex experimental design with measurement of the amount of feed consumed by individual animals will have a significant contribution to model improvement. A recent publication shows that the incorporation of drinking behavior in the population pharmacokinetic model of orally administered enrofloxacin in chickens leads to a significant improvement in the prediction of antibiotic concentration and the explanation of between-subject variability [31]. 

## 5. Conclusions

In conclusion, data from the current study of the pharmacokinetics of NAC in chickens provide initial information about the disposition of this drug after oral administration. NAC applied at a dose rate of 100 mg kg^−1^/24 h with the feed is absorbed at low concentrations without significant differences between healthy and *M. gallisepticum* chickens. The addition of a full dataset containing intravenous and oral NAC treatments in chickens, including higher doses, would help improve the model and our understanding of NAC pharmacokinetics in this animal species. Although our investigation attempted to calculate the obtained dose and to use it in pharmacokinetic modeling, the analysis indicates that this information was not sufficient to discover the source of variability between the individual animals. Incorporation of the behavior of the broilers and feed consumption could explain the between-subject variability.

## Figures and Tables

**Table 1:** Pharmacokinetic parameters (geometric mean ± geometric standard deviation, SD) in healthy broiler chickens (*n* = 6) after oral administration of N-acetylcysteine (received dose: 41 mg kg^−1^ bg/12 h).

Parameters	Units	Geometric Mean ± SD
k_ab_	h^−1^	0.67 ± 0.34
k_el_	h^−1^	0.21 ± 0.09
t_1/2ab_	h	1.04 ± 0.53
t_1/2el_	h	3.30 ± 1.43
T_max_	h	2.47 ± 0.45
C_max_	µg mL^−1^	2.26 ± 0.91
AUC_0-∞_	µg h mL^−1^	19.24 ± 4.25

T_max_: time of C_max_; C_max_: maximum plasma levels; k_ab_: absorption rate constant; k_el_: elimination rate constant; t_1/2ab_: absorption half-life; t_1/2el_: elimination half-life; AUC_0–∞_: area under the concentration–time curves from 0 to infinity ∞.

**Table 2:** Population parameters of orally administered N-acetylcysteine (targeted dose 50 mg/kg) in healthy broiler chickens (*n* = 12) and in broiler chickens infected with *Mycoplasma gallisepticum* (*n* = 20).

Parameters		Estimates	Units	SE	CV %	2.5% CI	97.5% CI
Groups treated solely with NAC							
Thetas (typical value)	tvka	0.26	1 h^−1^	0.04	15.61	0.18	0.34
	tvV	6.65	L kg^−1^	1.82	27.36	3.01	10.29
	tvk	0.51	1 h^−1^	0.10	19.28	0.32	0.71
	stdev0	0.47	µg/mL	0.04	8.71	0.39	0.56
All four groups							
Thetas (typical value)	tvka	0.142	1 h^−1^	0.025	17.65	0.093	0.192
	tvV	1.75	L kg^−1^	0.571	32.62	0.624	2.877
	tvk	1.219	1 h^−1^	0.306	25.10	0.615	1.823
	stdev0	0.349	µg/mL	0.025	7.13	0.301	0.399

Typical value (tv) of ka: absorption rate constant; V: volume of distribution; k: elimination rate constant; stdev0: standard deviation for multiplicative residual error; SE: standard error; CV: coefficient of variation; CI: Confidence interval.

**Table 3:** Random effects of orally administered N-acetylcysteine (targeted dose 50 mg/kg) in healthy broiler chickens (*n* = 12) and in broilers infected with *Mycoplasma gallisepticum* (*n* = 20).

Omega	Variance	SE	BSV (CV %)	Shrinkage
Groups treated solely with NAC				
ηka	0.023	0.002	15.09	0.155
ηk	0.057	0.013	24.15	0.176
ηV	0.371	0.066	67.05	0.156
All four groups				
ηka	0.593	0.177	60.66	0.351
ηk	0.576	0.327	88.21	0.356
ηV	1.14	0.572	145.78	0.252

Variance ηka, ηk, and ηV are random components of the model (Eta) and BSV is the between-subject variability estimated according to Equation (2), SE: standard error; BSV: between-subject variability; CV: coefficient of variation.

## Data Availability

Data are contained within the article and Supplementary Material.

## Supplementary Materials

The following are available online at https://www.mdpi.com/article/10.3390/vetsci8110244/s1, Figure S1: Plot of CWRES (conditional weighted residuals) against time after dose (a—groups treated solely with NAC; and b—data for all four groups); Table S1: Nutritional composition of the feed for broiler chickens administered during the treatment with NAC; Table S2: Plasma concentrations of N-acetylcysteine (NAC) in healthy broiler chickens treated with NAC through the feed for five consecutive days (received mean dose 41 mg kg^−1^ b.w./12 h); Table S3: Plasma concentrations of N-acetylcysteine (NAC) in healthy broiler chickens treated with NAC through the feed (received mean dose 47.5 mg kg^−1^ b.w./12 h) and doxycycline (20 mg kg^−1^ b.w./24 h via drinking water) for five consecutive days; Table S4: Plasma concentrations of N-acetylcysteine (NAC) in broiler chickens infected with Mycoplasma gallisepticum and treated with NAC through the feed for five consecutive days (received mean dose 43.29 mg kg^−1^ b.w./12 h); Table S5: Plasma concentrations of N-acetylcysteine (NAC) in broiler chickens infected with Mycoplasma gallisepticum and treated with NAC through the feed (received mean dose 43.3 mg kg^−1^ b.w./12 h) and doxycycline (20 mg kg^−1^ b.w./24 h) via drinking water for five consecutive days.
