# Supplementary material for: Absorption of N-acetylcysteine in Healthy and Mycoplasma gallisepticum-Infected Chickens
Source: Vet Sci. 2021 Oct 20;8(11):244. doi: 10.3390/vetsci8110244 (PMC8621408; doi:10.3390/vetsci8110244)
Supplement: Supplementary file 1 [file vetsci-08-00244-s001.zip › vetsci-1404236-supplementary.pdf]

veterinary sciences

MDPI

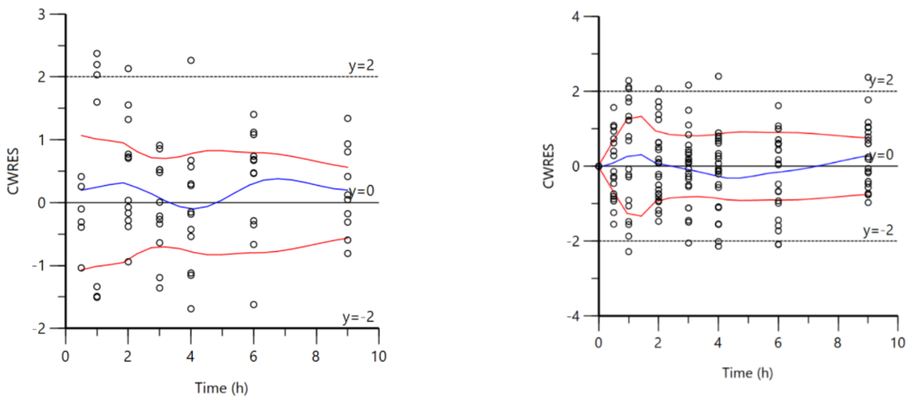

(**a**) (**b**)

Figure S1. Plot of CWRES (conditional weighted residuals) against time after dose (**a**, groups treated solely with NAC and **b**, data for all four groups).

Table S1. Nutritional composition of the feed for broiler chickens administered during the treatment with NAC.

Nutritional composition	%
Crude protein	18.95
Crude fat	6.71
Crude fiber	4.31
Crude ash	5.26
Calcium	0.75
Phosphorus	0.57
Sodium	0.16
Lysine	1.16
Methionine	0.54

Ingredients: corn, soybean meal, sunflower meal, sunflower oil, sodium chloride, L-lysine hydrochloride, DL-methionine, monocalcium phosphate, calcium carbonate, L-threonine; Supplements: Supplied per kg diet: Fe 109.58 mg, Cu 7.05 mg, Zn 31.66 mg, Mn 15.76 mg, iodine 0.08 mg, selenium 0.02 mg, retinol acetate 10000 UI, cholecalciferol 4000 UI, choline chloride 402 mg.

Table S2. Plasma concentrations of N-acetylcysteine (NAC) in healthy broiler chickens treated with NAC trough the feed for five consecutive days (received mean dose 41 mg.kg^{-1} bw/12 h).

Time (h)	1	2	3	4	5	6	Mean±SD
0.5	<LOQ	0.63	0.41	0.70	0.71	0.37	0.50±0.20
1	0.19	0.19	2.21	3.38	3.56	3.09	2.10±1.55
2	2.74	2.13	1.66	4.26	3.32	1.65	2.63±1.03
3	0.44	1.78	1.54	3.19	3.75	2.56	2.21±1.20
4	0.91	1.53	3.64	1.78	3.06	2.16	2.18±1.01
6	1.10	0.82	0.88	1.44	2.75	1.85	2.12±1.84
9	0.57	0.75	0.72	0.83	1.75	1.08	0.95±0.43
12	2.61	0.94	1.85	3.38	1.35	3.60	2.29±1.08
14	1.43	0.80	1.23	1.81	3.35	2.60	1.87±0.95
24	2.78	2.70	1.34	1.40	2.72	1.74	2.11±0.70
120	3.10	0.00	1.10	2.57	1.60	1.17	1.59±1.11
122	1.92	<LOQ	<LOQ	1.63	1.00	1.66	1.09±0.79
124	<LOQ	<LOQ	<LOQ	<LOQ	<LOQ	<LOQ	<LOQ
126	<LOQ	<LOQ	<LOQ	<LOQ	<LOQ	<LOQ	<LOQ
132	<LOQ	<LOQ	<LOQ	<LOQ	<LOQ	<LOQ	<LOQ
144	<LOQ	<LOQ	<LOQ	<LOQ	<LOQ	<LOQ	<LOQ
152	<LOQ	<LOQ	<LOQ	<LOQ	<LOQ	<LOQ	<LOQ
168	<LOQ	<LOQ	<LOQ	<LOQ	<LOQ	<LOQ	<LOQ
174	<LOQ	<LOQ	<LOQ	<LOQ	<LOQ	<LOQ	<LOQ

<LOQ – the concentration is below the limit of quantification for the used method.

Table S3. Plasma concentrations of N-acetylcysteine (NAC) in healthy broiler chickens treated with NAC trough the feed (received mean dose 47.5 mg.kg^{-1} bw/12 h) and doxycycline (20 mg.kg-1 bw/24 h via drinking water) for five consecutive days.

Time (h)	1	2	3	4	5	6	Mean±SD
0.5	2.99	3.01	1.78	0.95	1.25	0.35	1.72±1.09
1	1.75	2.35	2.25	2.15	1.85	1.88	2.04±0.24
2	1.61	1.58	1.85	2.84	1.99	2.88	2.13±0.59
3	1.21	2.14	2.43	2.07	2.29	1.08	1.87±0.58
4	1.38	2.45	2.28	2.83	0.88	0.94	1.80±0.83
6	1.02	1.94	0.59	0.66	0.83	0.74	0.96±0.50
9	0.41	1.86	<LOQ	1.04	1.12	1.90	1.27±0.62
12	0.84	1.46	0.60	1.94	2.68	2.46	1.66±0.85
14	1.01	3.20	0.58	2.75	3.09	2.39	2.17±1.11
24	1.38	0.04	1.26	1.12	2.87	2.43	1.51±1.01
120	4.09	8.33	1.49	<LOQ	2.26	2.45	3.72±2.74
122	2.42	0.35	0.84	<LOQ	<LOQ	0.48	1.02±0.95
124	0.38	<LOQ	<LOQ	<LOQ	<LOQ	<LOQ	0.38±0.00
126	<LOQ	<LOQ	<LOQ	<LOQ	<LOQ	<LOQ	<LOQ
132	<LOQ	<LOQ	<LOQ	<LOQ	<LOQ	<LOQ	<LOQ
144	<LOQ	<LOQ	<LOQ	<LOQ	<LOQ	<LOQ	<LOQ
152	<LOQ	<LOQ	<LOQ	<LOQ	<LOQ	<LOQ	<LOQ
168	<LOQ	<LOQ	<LOQ	<LOQ	<LOQ	<LOQ	<LOQ
174	<LOQ	<LOQ	<LOQ	<LOQ	<LOQ	<LOQ	<LOQ

<LOQ – the concentration is below the limit of quantification for the used method.

Table S4. Plasma concentrations of N-acetylcysteine (NAC) in broiler chickens infected with Mycoplasma gallisepticum and treated with NAC trough the feed for five consecutive days (received mean dose 43.29 $mg.kg^{-1}$ bw/12 h).

Time (h)	1	2	3	4	5	6	7	8	9	10	Mean±SD
0.5	<LOQ	-	<LOQ	-	<LOQ	-	<LOQ	<LOQ	-	0.33	0.33±0.00
1	0.32	<LOQ	-	<LOQ	-	<LOQ	-	-	<LOQ	<LOQ	0.32±0.00
2	-	1.26	1.43	-	1.62	2.34	0.88	-	2.40	-	1.66±0.60
3	0.31	-	1.29	1.12	-	-	1.12	1.58	-	1.84	1.21±0.52
4	-	1.25	-	1.20	2.04	-	-	0.76	0.30	0.63	1.03±0.61
6	1.26	1.33	1.63	-	0.32	1.57	-	-	1.49	-	1.26±0.48
9	<LOQ	-	-	0.36	-	0.60	0.46	0.31	-	0.62	0.47±0.14
12	-	0.57	1.14	-	4.92	-	2.02	-	2.84	3.80	2.55±1.64
14	-	1.00	1.18	0.72	-	0.98	-	0.66	1.12	-	0.94±0.21
24	1.68	-	-	2.08	1.88	-	1.47	1.61	-	<LOQ	1.74±0.24
120	-	1.45	1.32	-	0.70	0.97	<LOQ	-	0.65	-	1.02±0.36
122	0.61	-	0.63	<LOQ	-	0.96	-	0.54	-	0.32	0.61±0.23
124	<LOQ	<LOQ	-	<LOQ	<LOQ	-	<LOQ	-	<LOQ	-	<LOQ
126	-	<LOQ	<LOQ	-	-	<LOQ	-	<LOQ	<LOQ	<LOQ	<LOQ
132											
144											
152											
168						<LOQ					
174											

- – concentrations were not determined (blood samples were not taken from the chicken at the given time interval); <LOQ – the concentration is below the limit of quantification for the used method.

Table S5. Plasma concentrations of N-acetylcysteine (NAC) in broiler chickens infected with Mycoplasma gallisepticum and treated with NAC trough the feed (received mean dose 43.3 mg.kg^{-1} bw/12 h) and doxycycline (20 mg.kg-1 bw/24 h via drinking water) for five consecutive days.

Time (h)	1	2	3	4	5	6	7	8	9	10	Mean±SD
0.5	2.44	-	2.00	-	3.84	-	1.73	0.63	-	2.30	2.15±1.05
1	1.76	4.54	-	7.34	-	1.59	-	-	4.19	4.02	3.91±2.11
2	-	4.95	1.75	-	3.80	1.43	3.09	-	6.36	-	3.56±1.89
3	2.37	-	2.32	8.15	-	-	2.71	1.68	-	7.05	3.44±2.66
4	-	1.54	-	6.85	1.63	-	-	2.09	2.77	4.04	3.15±2.03
6	0.94	0.57	2.15	-	2.15	1.20	-	-	3.15	-	1.69±0.96
9	1.07	-	-	5.60	-	1.04	1.91	1.44	-	2.81	2.31±1.74
12	-	1.00	3.30	-	1.22	-	1.43	-	4.84	4.39	2.70±1.70
14	-	1.23	1.10	2.51	-	1.64	-	<LOQ	3.92	-	2.08±1.17
24	1.38	-	-	2.70	1.42	-	2.24	1.80	-	3.69	1.91±0.56
120	-	0.83	<LOQ	-	0.36	0.41	0.56	-	0.90	-	0.61±0.24
122	0.68	-	0.48	<LOQ	-	0.56	-	0.67	-	<LOQ	0.60±0.10
124	<LOQ	<LOQ	-	<LOQ	<LOQ	-	<LOQ	-	<LOQ	-	<LOQ
126	-	<LOQ	<LOQ	-	-	<LOQ	-	<LOQ	<LOQ	<LOQ	<LOQ
132	<LOQ										
144											
152											
168											
174											

- – concentrations were not determined (blood samples were not taken from the chicken at the given time interval); <LOQ – the concentration is below the limit of quantification for the used method
